# Yeast Biosensors for the Safety of Fermented Beverages

**DOI:** 10.3390/bios16010064

**Published:** 2026-01-16

**Authors:** Sílvia Afonso, Ivo Oliveira, Alice Vilela

**Affiliations:** 1 Centre for the Research and Technology of Agroenvironmental and Biological Sciences (CITAB), Institute for Innovation, Capacity Building and Sustainability of Agri-Food Production (Inov4Agro), University of Trás-os-Montes and Alto Douro (UTAD), Quinta de Prados, 5000-801 Vila Real, Portugal; safonso@utad.pt; 2 Centre for the Research and Technology of Agroenvironmental and Biological Sciences (CITAB), Institute for Innovation, Capacity Building and Sustainability of Agri-Food Production (Inov4Agro), Department of Agronomy, School of Agrarian and Veterinary Sciences, University of Trás-os-Montes and Alto Douro (UTAD), Quinta de Prados, 5000-801 Vila Real, Portugal; ivo.vaz.oliveira@utad.pt; 3 Chemistry Research Centre-Vila Real (CQ-VR), Department of Agronomy, School of Agrarian and Veterinary Sciences, University of Trás-os-Montes and Alto Douro (UTAD), 5001-801 Vila Real, Portugal

**Keywords:** toxins, biogenic amines, heavy metals, preservatives and additives, fermentation byproducts, endocrine-disrupting chemicals, spoilage microorganisms

## Abstract

Yeast biosensors represent a promising biotechnological innovation for ensuring the safety and quality of fermented beverages such as beer, wine, and kombucha. These biosensors employ genetically engineered yeast strains to detect specific contaminants, spoilage organisms, or hazardous compounds during fermentation or the final product. By integrating synthetic biology tools, researchers have developed yeast strains that can sense and respond to the presence of heavy metals (e.g., lead or arsenic), mycotoxins, ethanol levels, or unwanted microbial metabolites. When a target compound is detected, the biosensor yeast activates a reporter system, such as fluorescence, color change, or electrical signal, providing a rapid, visible, and cost-effective means of monitoring safety parameters. These biosensors offer several advantages: they can operate in real time, are relatively low-cost compared to conventional chemical analysis methods, and can be integrated directly into the fermentation system. Furthermore, as *Saccharomyces cerevisiae* is generally recognized as safe (GRAS), its use as a sensing platform aligns well with existing practices in beverage production. Yeast biosensors are being investigated for the early detection of contamination by spoilage microbes, such as *Brettanomyces* and lactic acid bacteria. These contaminants can alter the flavor profile and shorten the product’s shelf life. By providing timely feedback, these biosensor systems allow producers to intervene early, thereby reducing waste and enhancing consumer safety. In this work, we review the development and application of yeast-based biosensors as potential safeguards in fermented beverage production, with the overarching goal of contributing to the manufacture of safer and higher-quality products. Nevertheless, despite their substantial conceptual promise and encouraging experimental results, yeast biosensors remain confined mainly to laboratory-scale studies. A clear gap persists between their demonstrated potential and widespread industrial implementation, underscoring the need for further research focused on robustness, scalability, and regulatory integration.

## 1. Introduction

In recent decades, there has been a significant increase in food contamination cases, accompanied by growing concern among consumers and authorities regarding food quality and safety [1]. According to the World Health Organization [2], contaminated food is responsible for approximately 600 million cases of foodborne illnesses and 420,000 deaths annually worldwide. This scenario is driven not only by the globalization of the food supply chain and the intensive use of pesticides and additives, but also by failures in control systems and technological limitations in the rapid detection of contaminants [3]. Food is often contaminated with microbial toxins produced by bacteria, parasites, fungi, and viruses [4]. Ensuring safe food with both nutritional and sensory quality has become a public health priority, particularly for high-consumption products, such as fermented beverages [5,6,7].

Food fermentation is the second most used food processing method, reflecting variations in the food substrate, cultural influences, and microbial biodiversity [8]. These processes alter the organoleptic and nutritional properties of the products, resulting in new functional characteristics [9,10]. In the context of fermented beverages, including traditional ones such as tepache [11] and khadi [12], as well as widely consumed beverages like wine, beer, kombucha, and kefir, these transformations are significant and influence both the sensory quality and safety of the product [10,13], even though the negative health effect of alcohol is well established [14]. These products, enjoyed globally, not only drive the strategic agri-food sector but also align with market trends in health and well-being. However, their fermentative nature and the complexity of the food matrix make them susceptible to contamination by undesirable compounds throughout the production process [10,15].

Among the most concerning contaminants in these beverages are mycotoxins, such as aflatoxins and ochratoxin A, associated with fungal contamination by *Aspergillus* and *Penicillium* in grapes and cereals; biogenic amines, such as histamine, tyramine, and putrescine; heavy metals, such as lead, cadmium, and arsenic, originating from soil, water, or production utensils; pesticide residues, resulting from the intensive use of agrochemicals in viticulture and cereal production; and pathogenic microorganisms such as some virulent strains of *Escherichia coli* [16,17], *Salmonella*, and *Listeria monocytogenes*, which can compromise microbiological quality and pose a direct health risk to consumers [10,18,19,20]. Considering their potential health risks, maximum legal limits for these contaminants have been established by international regulations.

Endocrine-disrupting chemicals (EDCs) are another contaminant to consider. They are widespread pollutants that interfere with hormone systems even at very low doses. Research links them to reproductive, metabolic, neurodevelopmental, and cancer risks, mainly when exposure occurs during fetal life, childhood, or pregnancy. There is active debate over whether EDCs have thresholds below which exposure is safe: Some toxicology studies on specific pesticides find clear thresholds of adverse effects, suggesting that reference doses can be set [21,22]; Endocrinology-focused groups argue that non-monotonic, low-dose and developmental effects make standard threshold-based approaches unreliable, especially for pregnancy and childhood [23,24]. Unfortunately, policies differ: the EU increasingly uses a hazard-based approach (minimizing exposure and banning some uses), while the US and others mainly use risk-based limits that many authors consider too slow and incomplete [25].

Fermented beverages can carry antibiotic-resistant microbes and genes, so the central “limit” is strict control of contamination, not the addition of antibiotic doses. The main concerns are antibiotic-resistant bacteria and genes (ARGs). Fermented foods and beverages (dairy and non-dairy) often contain lactic acid bacteria and other microbes that harbor ARGs to tetracyclines, penicillins, macrolides, and others [26,27]. Metagenomic surveys show a high prevalence of ARGs in many traditional fermented foods (kimchi, artisan cheeses, kefir), some of which carry clinically important resistance and transferable genes [28].

In dairy chains, routine antibiotic use in livestock and mastitis treatment increases ARGs and resistant bacteria in raw milk; experimental work shows plasmid-mediated ARG transfer between milk bacteria [26]. Antibiotic residues in process water can enter the wine fermentation process; experimental contamination with ivermectin, ciprofloxacin, azithromycin, or hydroxychloroquine inhibited yeast metabolism and reduced polyphenol diffusion by 40–50%, altering wine quality and implying selection pressure on microbes [29].

Table 1 summarizes the current regulatory thresholds for fermented beverages.

Despite their high sensitivity and reliability, conventional analytical methods used for detecting these contaminants, such as high-performance liquid chromatography (HPLC), gas chromatography coupled with mass spectrometry (GC-MS), and enzyme-linked immunosorbent assays (ELISA), have limitations that must be considered. These techniques are typically expensive, require sophisticated equipment and skilled technical personnel, and are not readily applicable in situ or in resource-limited environments [7,10,55].

In this context, biosensors emerge as a promising solution, offering rapid, cost-effective, sensitive, efficient, and specific responses [56]. These devices combine biological recognition elements, such as enzymes, antibodies, DNA, or living cells, with physicochemical transducers, enabling real-time detection of various analytes. Their miniaturization, multiplexing capability, and potential for integration with mobile technologies, intelligent systems, and machine learning algorithms make them particularly attractive for monitoring contaminants throughout the production chain of fermented beverages [15,55,56].

Among microbial biosensors, yeast-based biosensors (YBB) have attracted growing interest, even though their share with their bacterial counterparts is their stability, robustness, low cost, tolerance to diverse measurement conditions, and high potential for genetic modification to enhance specificity [57,58,59], mostly due to their “eukaryotic advantage” (discussed further in the next chapter). They employ viable or non-viable cells as sensing elements, exploiting either their natural metabolic activity or genetically engineered reporter systems—such as fluorescence, luminescence, colorimetry, or electrochemical signals. Advances in synthetic biology, nanotechnology, and the incorporation of heterologous receptors, such as G-protein-coupled receptors, have expanded the capabilities of YBB, enabling the detection of a wide range of chemical and microbiological hazards relevant to fermented beverages [57,60].

A special case of study is the Yeast Estrogen Screen (YES) and Yeast Androgen Screen (YAS), which are standard whole-cell assays that use genetically modified *Saccharomyces cerevisiae* expressing human hormone receptors to detect estrogenic and androgenic endocrine-disrupting compounds (EDCs). They are widely used for screening chemicals and environmental samples because they are low-cost, robust, and report actual receptor-mediated activity [61,62].

Research also shows that engineered yeast can reliably detect tetracycline antibiotics (especially doxycycline) and at least one azole fungicide in tablets, liquids, and biological fluids, often on low-cost paper strips [63,64].

Thus, this review aims to provide a comprehensive overview of yeast-based biosensors for ensuring the safety of fermented beverages, discussing their operating principles, recent technological advances, ability to detect chemical and microbiological contaminants, advantages over conventional methods, current limitations, and prospects for real-world application.

## 2. Yeast Biosensors: Principles and Design

### 2.1. Definition and General Architecture of Biosensors

**Figure 1:**
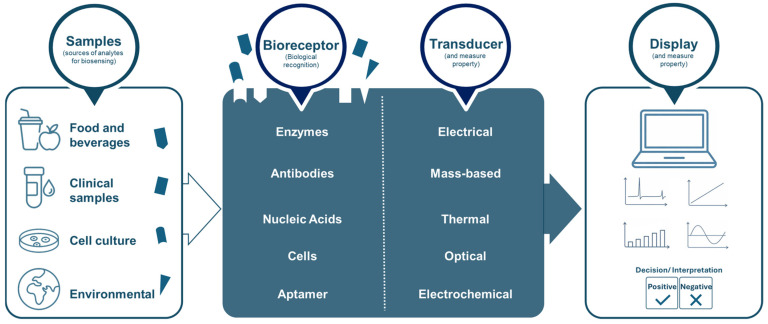
General schematic of a biosensor. Samples (food and beverages, clinical samples, cell cultures, and environmental samples) interact with a bioreceptor (enzymes, antibodies, nucleic acids, cells, or aptamers). A transducer converts this specific biological recognition into measurable signals (electrical, mass-based, thermal, optical, or electrochemical), which are then processed and presented by the display system to enable interpretation and decision-making—figure made by the authors.

Biosensors are a novel accomplishment within analytical biotechnology, being defined by IUPAC as a device that uses specific biochemical reactions mediated by isolated enzymes, immunosystems, tissues, organelles, or whole cells to detect chemical compounds, frequently by electrical, thermal, or optical signals [65]. Structurally, biosensors are composed of a biological recognition component able to sense or interact with target molecules, combined with a physicochemical transducer and a microelectronic processor. Together, these compounds amplify and convert the biological response into a readable signal [15], as shown in Figure 1.

Compared with more complex analytical techniques such as gas chromatography, liquid chromatography, and mass spectrometry, biosensors offer a simpler, more cost-effective, and compact alternative. Their small size and low production cost make them particularly suitable for integration into extensive sensor networks [58,59,66,67]. However, the critical maintenance requirements and limited lifetimes severely hinder their integration into hardware platforms [68].

Microorganisms, both bacteria or yeasts and microalgae, are studied as biological recognition elements, combining the biological receptor and the transducer in one component, with interactions with the target compound resulting in information expressed by changes in physiological, morphological, or biochemical parameters (e.g., cell yield, growth rate, shape, permeability, survival, and metabolism) [59]. The use of this type of organism has several advantages, including the indication of analyte bioavailability, a self-sustaining biorecognition element, user-friendliness, and low cost [58,69].

Among these microbial biorecognition systems, yeasts are particularly valuable candidates due to their distinctive biological attributes and suitability for biosensing applications. There are several advantages to using yeast, cleverly defined by Walmsley and Keenan [70]. First, yeasts share the same “microbial advantages” that bacteria provide, including speed of growth, easy manipulation, and growth on a variety of different carbon sources. Second, they present the “eukaryotic advantage”: yeasts share cellular features and molecular mechanisms with human cells, which are highly relevant to sensing and responding to environmental stimuli. Third, the “yeast advantage”, referring to microorganisms that are particularly strong within a range of physicochemical tolerance. Finally, the “*Saccharomyces* advantage”, when referring to this specific yeast, one of the best understood and manipulated organisms, is a model for providing knowledge for higher eukaryotes, particularly at gene function analysis [70]. In addition, many laboratory yeast species are classified as Generally Recognized as Safe (GRAS) by the US Food and Drug Administration (FDA), reinforcing their suitability as safe, reliable, and industry-relevant biological systems for biosensor development [71]. Moreover, improved microbial genetic manipulation, enhanced by “omics” technologies, metabolic engineering, and in silico modeling, now offers new opportunities for creating more accurate and sensitive biosensors based on microbial cells [59].

In cell-based biosensing, two major design paradigms are commonly distinguished: transcription-dependent biosensors and transcription-independent biosensors. Transcription-dependent biosensors rely on genetically encoded regulatory circuits in which the presence of a target ligand modulates transcriptional activity, typically via a sensory protein such as a transcription factor (TF) that binds the analyte and then activates or represses expression of a measurable reporter gene (e.g., fluorescent proteins or enzymes). These systems translate ligand recognition into changes in gene expression, enabling quantitative readouts of metabolite or chemical concentrations in vivo [72]. In contrast, transcription-independent biosensors do not depend on de novo gene expression to generate signals. Instead, they convert binding or metabolic transformation of the analyte into easily detectable outputs through alternative pathways, such as metabolic production of a proxy signal molecule or direct conformational changes in molecular recognition elements that are transduced into optical, electrical, or other signals. This approach can circumvent limitations of transcriptional regulation, particularly when the target molecule is difficult to sense via genetic circuits, and has been explicitly described in the context of yeast biosensing, where metabolic byproducts or post-translational systems are leveraged for detection [57].

### 2.2. Advantages of Yeast-Based Systems

Whole-cell yeast biosensors can provide specific detection of compounds without pretreatment, an advantage over enzyme or aptamers, and, given the growth conditions required for yeasts, they are more suitable for on-site applications and potentially adaptable to portable devices for in situ testing [57,60].

**Figure 2:**
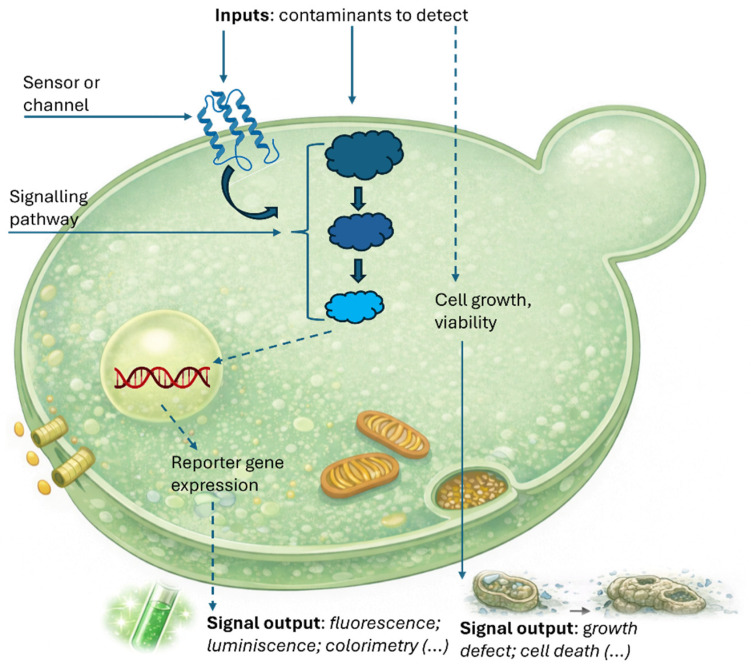
Illustration of the different key features of yeast cells used as sensitive elements in current biosensors. Figure adapted from Martin-Yken et al. [60].

Yeast cells can either perform sensing and signal transduction intrinsically or be genetically engineered for this purpose, with genetic modification being the more commonly employed strategy. Their activity is modulated through direct or indirect interactions with target analytes, producing optical signals detectable as colorimetric, fluorometric, or luminometric changes. Reporter gene expression may be constitutive or inducible. In constitutive systems, the presence of the analyte indirectly affects reporter expression by influencing cellular parameters such as growth, survival, or metabolism [58,59]. The design of inducible reporter genes generally follows two main strategies: (i) the target compound binds to a regulatory protein, inhibiting reporter gene expression (negative regulation), or (ii) it interacts with a receptor protein—either membrane-bound or intracellular—thereby activating gene expression (positive regulation). Inducible sensor strains offer greater specificity than constitutive systems, as the target analyte directly modulates their response, with the response proportional to its concentration. Sensitivity is further enhanced by intracellular transcriptional and translational cascades triggered by analyte binding [59]. In Figure 2, we illustrate the principal features of yeast cells as biosensor components. Specific environmental contaminants can permeate the yeast cell membrane, directly influencing cellular growth and viability. In contrast, larger molecules typically remain extracellular. Nevertheless, these larger entities can still be detected via membrane-bound sensor proteins or channels, which relay signals to intracellular signaling cascades and transcription factors. The resultant outputs may manifest as direct impacts on cellular proliferation or viability, or as more indirect pathways facilitated by enzymatic activities that often, but not exclusively, involve modulation of gene expression [60].

### 2.3. Reporter Systems and Signal Transduction

Numerous YBB reporters have been developed and applied in scientific research to investigate key cellular mechanisms. These systems predominantly rely on reporter gene expression regulated by inducible promoters, which are activated by corresponding transcription factors under specific conditions [57]. Yeast biosensor outputs may be quantified using diverse detection approaches, including fluorescence, luminescence, enzymatic activity (typically detected via substrate color change), electrical signals, or variations in growth rate [57,60], Figure 2. The main classes of yeast-based biosensors and their corresponding detection principles are summarized in Table 2. However, most YBBs remain in the early developmental stage, and only a few prototypes have been tested in real applications [73].

#### 2.3.1. Fluorescent and Luminescent Reporters

Fluorescent sensing is based on measuring fluorescence intensity and correlating it to the concentration of the target analyte. Fluorescent biosensors are widely used in analytical chemistry due to their straightforward construction using standard molecular biology techniques [75]. These biosensors are categorized as in vivo and in vitro. In in vivo systems, microorganisms endogenously produce fluorescent proteins (e.g., GFP) without the need for external fluorescent agents. In in vitro systems, microbial metabolic activity alters the surrounding environment, affecting the emission of externally added fluorescent compounds.

Fluorescent (and luminescent) biosensors often use enzymes such as *LacZ* (β-galactosidase) to convert weak biological signals into strong optical readouts. Recent work combines classical *LacZ* systems with modern fluorescent proteins and advanced fluorogenic substrates to improve sensitivity, single-cell resolution, and in vivo applicability. Classical LacZ is widely used in whole-cell biosensors that employ chromogenic/fluorogenic or chemiluminescent substrates for environmental and drug-screening applications [81]. *LacZα*-complementation systems greatly boost sensitivity: small *lacZα* fragments driven by test promoters complement a chromosomally encoded ω-fragment, allowing detection of very low transcription levels and outperforming mCherry fluorescence [82,83]. A dual mCherry–lacZα bicistronic reporter enables simultaneous fluorescence imaging and high-gain enzymatic readout in lead biosensing; the lacZα arm is more sensitive than both full-length LacZ and mCherry [82].

New fluorogenic β-gal substrates (e.g., SPiDER βGal, single-cell probes) become strongly fluorescent upon cleavage, are retained within cells, and enable live-tissue and single-cell LacZ imaging as well as multiparameter flow cytometry [84,85].

Bioluminescent biosensors, by contrast, detect luminescence emitted by microorganisms in response to the analyte in a dose-dependent manner. Unlike fluorescence, which measures protein abundance, bioluminescence quantifies enzymatic activity, enabling faster and more sensitive detection [74]. Fluorescence-based reporters include Green Fluorescent Protein (GFP), Red Fluorescent Protein (RFP), and Yellow Fluorescent Protein (YFP). In contrast, luminescence reporters include Bacterial Luciferase (Lux) and Firefly Luciferase (Luc), both of which offer a significant advantage: (i) Lux (bacterial luciferase/luxCDABE) can be fully autonomous: luciferase + luciferin synthesis encoded in one operon, no exogenous substrate needed [76], enabling continuous real-time monitoring in vitro and in vivo [86]. However, output is typically weaker than firefly luciferase and often requires longer integration times for animal imaging [86,87]; (ii) Firefly Luc (*Photinus pyralis* and related) requires exogenous D-luciferin, ATP, Mg^2+^, giving very high photon output and sensitivity [88].

#### 2.3.2. Enzymatic, Colorimetric, and Electrochemical Reporters

Remarkably, most YBB developed in recent years employ optical transduction mechanisms with fluorescence, luminescence, or color change serving as the primary output signals. Enzymatic and colorimetric biosensors rely on enzyme-mediated reactions that produce a visible color change or a measurable absorbance variation, often using β-galactosidase or peroxidase as reporter enzymes. These systems are appreciated for their simplicity, low cost, and suitability for visual or spectrophotometric detection, although their quantitative precision may be limited [60,69]. Yeast-based enzymatic biosensors have also been applied to the monitoring of fermentation-related metabolites such as ethanol, glucose, and lactate, demonstrating their potential for real-time quality control in alcoholic beverages [77,78,79].

However, the development of yeast biosensors employing electrical reporters, such as amperometric detection, should be considered given their advantages in sensitivity, equipment cost, and compatibility with portable devices [79]. In amperometric biosensors, detection is based on measuring changes in electric current resulting from specific cellular reactions and facilitated by specialized electrodes. Other approaches to electrical reporters include conductometric biosensors, which provide fast and highly sensitive responses to analytes; potentiometric biosensors, which detect the analytes by measuring the potential difference between a working electrode and a reference electrode separated by a selective membrane; and voltammetric approaches such as cyclic voltammetry [80]. In the context of fermented beverages, electrochemical biosensors based on *Saccharomyces cerevisiae* or *Hansenula polymorpha* have been developed for ethanol determination and process monitoring, offering rapid response and high sensitivity [77].

## 3. Detection Targets in Fermented Beverages

### 3.1. Chemical Contaminants

#### 3.1.1. Heavy Metals

Several targets can be achieved by using YBB in fermented beverages. One of such targets are heavy metals, which represent a significant threat to human health. They are particularly suitable targets for biosensor development, as many cell types are susceptible to metal exposure and possess well-conserved pathways that mediate rapid and robust responses to metal-induced stress [60]. Metals play a crucial role in biological systems, since several function as essential cofactors for enzymes involved in cellular metabolism and growth. In the absence of these important trace metals, fundamental life processes would not be possible. However, certain metals—particularly those classified as “heavy metals”—are also among the most abundant, toxic, and persistent inorganic environmental pollutants [89].

Cellular responses to heavy metals often involve metal-chelating molecules such as glutathione, phytochelatins, and metallothioneins. The promoters of genes involved in these pathways are strongly inducible by heavy metals and serve as key regulatory elements for the construction of highly sensitive and specific biosensors. Alternatively, promoters of genes encoding antioxidant enzymes—such as superoxide dismutases, catalases, and glutathione peroxidases—may also be used, given their inducibility by metal ions [90], even though issues with specificity may arise, as more than one ion may induce a response [90].

Only a few examples exist of the use of yeasts for the detection of heavy metals. A system employing *S. cerevisiae* cells with green fluorescent protein (*GFP*) as the reporter was developed for the detection of copper ions [91]. This biosensor utilizes the yeast’s native transcriptional activator Ace1 to regulate *GFP* expression in response to Cu^2+^. It can detect copper at concentrations as low as 0.5 µM, exhibiting high selectivity for Cu^2+^ over other metal ions, except Ag^+^, which also induces a response [91]. Using the same promoter (*CUP1*) but a different reporter gene (luciferase), other authors also achieved a biosensor with a similar limit of detection of about 0.5 M for Cu^2+^ ions [92]. Further applications of *CUP1*, with *LacZ* or *Ade2* as reporter genes, have also been reported for copper detection [93]. In fermented beverages, the presence of heavy metals such as Cu^2+^, Cd^2+^, Pb^2+^, or As^3+^ may arise from raw materials, water, or processing equipment, making their rapid detection essential for quality control. YBB offers a suitable approach, as their metal-responsive promoters are naturally inducible within the concentration ranges relevant to these matrices.

More recent research has expanded the use of *S. cerevisiae* for copper detection through several approaches [59,91], including a combined dual-reporter fluorescent system with an engineered *Cup1* promoter and overexpressed *Cup2* transactivator, which was able to achieve a maximum linear range of 10^−8^ to 10^−3^ M of bioavailable copper [94].

For cadmium detection, a reporter construct was developed using the *HSEO1* gene promoter and the *GFP* gene, which responded in a dose-dependent manner, with Cd detection ranging from 1 μM to 900 μM; arsenic also elicited a response to a lesser extent [95]. Additionally, a system combining *S. cerevisiae*, *Escherichia coli*, and *Bacillus subtilis* was developed to detect both Cu^2+^ and Cd^2+^ ions [96]. For cadmium detection, the use of a 500 bp *HpSEO1* promoter from yeast *Hansenula polymorpha* directed specific expression of *GFP* upon exposure to Cd in a dose-dependent manner, with Cd detection ranging from 1 to 900 μM [95]. Yeast-based biosensors have been reported to detect heavy metal contaminants ranging from 5 × 10^−7^ M to 0.5 mM [62,63,96,97,98,99], indicating their ability to provide detection below the defined thresholds.

#### 3.1.2. Mycotoxins

Another chemical target for YBB in fermented beverages are mycotoxins. They are the primary contaminants of food and feed worldwide, and mycotoxins include a wide array of compounds produced by fungi, mainly from the *Aspergillus*, *Penicillium*, and *Fusarium* genera. Because grapes, cereals, and other raw materials used in fermented beverages are highly susceptible to fungal contamination before processing, mycotoxins are particularly relevant hazards in wine, beer, kombucha, and other fermented drinks.

One of the earliest attempts to develop a yeast-based biosensor for mycotoxin detection was made in 1984. Using the yeast *Kluyveromyces fragilis*, Schappert & Khachatourians [100] designed a system capable of detecting T2 toxin, which also worked for verrucarin A [100]. This assay was further refined using *Kluyveromyces marxianus*, where inhibition of β-galactosidase activity served as the detection principle [101]. Although these early assays were not explicitly developed for fermented beverage matrices, they demonstrated that yeast-based systems could respond to toxic fungal metabolites commonly found in cereals and fruit substrates used in beverage fermentation.

Zearalenone (ZON) is an important non-steroidal estrogenic mycotoxin produced by *Fusarium* species. It is particularly relevant to beer and other cereal-based beverages. Researchers engineered a strain of *Saccharomyces cerevisiae* to express the human estrogen receptor (*hER*), allowing the yeast to grow only when ZON activated the receptor [102]. This strain was later enhanced to include a firefly luciferase enzyme, which emits light upon exposure to a D-luciferin substrate [103]. This type of estrogen-responsive yeast biosensor is highly significant for beer production, as ZON commonly contaminates barley and other cereals prior to malting.

Yeast biosensors have also been engineered for the detection of aflatoxin. *S. cerevisiae* strains expressing reporter constructs responsive to Aflatoxin B1 (*AFB1*) have been developed [104]. Another approach to mycotoxins, namely aflatoxins B1 (*AFB1*) and M1 (*AFM1*), was achieved by β-galactosidase activity in *S. cerevisiae* [105]. These approaches are relevant not only to cereal-based beverages but also to wine, where aflatoxin contamination may occur in grapes exposed to *Aspergillus flavus* during pre- or post-harvest.

Deoxynivalenol (DON), or vomitoxin, is another common mycotoxin found in cereal crops and their products. The study by Yang et al. [106] describes the construction of a Fab antibody library displayed on the surface of *Saccharomyces cerevisiae* using yeast surface display (YSD) for the detection of the mycotoxin deoxynivalenol (DON). MACS/FACS isolated a yeast subpopulation with high specificity and subsequently employed it to develop a Fab@YSD-based chemiluminescent biosensor exhibiting high sensitivity and a wide linear response range for DON. The use of yeast-based biosensors to detect mycotoxins below their legal limits is feasible and has been reported previously [107].

#### 3.1.3. Biogenic Amines

Biogenic amines are low-molecular-weight compounds formed by microbial decarboxylation of amino acids, and in fermented beverages, they can accumulate during fermentation and storage, leading to potential toxicological effects in consumers [108]. Several biosensors can detect biogenic amines [109]. One example is a *Pseudomonas putida* whole-cell biosensor expressing a fluorescent protein that achieved a detection limit of 0.39 ppm for histamine, with reduced reactivity toward other biogenic amines or amino acids [110].

To the best of our knowledge, no yeast-based biosensors have yet been reported for the specific detection of biogenic amines in fermented beverages. This area remains largely unexplored despite its significant potential to enhance quality control in these matrices. Biosensor platforms for biogenic amines—such as enzymatic or molecularly imprinted systems—have demonstrated limits of detection in the micromolar range for histamine and tyramine (e.g., 7.5 × 10^−6^ M to 1.46 × 10^−5^ M), which match regulatory and spoilage thresholds [111].

#### 3.1.4. Fermentation By-Products (Acetaldehyde, Acetic Acid, Among Others)

Acetaldehyde, a naturally occurring compound from the aldehyde group, is produced during cellular metabolism and is a key intermediate in alcoholic fermentation. Although it is considered safe (GRAS) and is widely used as a flavoring agent [112], the International Agency for Research on Cancer (IARC) classifies acetaldehyde as a Group 1 carcinogen, indicating confirmed carcinogenicity in humans. Its toxicological effects include DNA damage, enzyme inactivation, and disruption of cellular homeostasis [113].

In winemaking, acetaldehyde plays a pivotal role in determining sensory characteristics, as at low concentrations it contributes desirable, fruity, fresh notes. In contrast, elevated levels are associated with off-flavors often described as green, vegetal, or bruised apple [114,115]. Despite its importance, few studies have focused on the design of biosensors for acetaldehyde detection. Previous studies explored alternative approaches for acetaldehyde detection, including nanomaterial-based sensing platforms, electrochemical biosensors with enhanced sensitivity, and conventional analytical techniques such as gas and liquid chromatography [116,117,118,119]. Even so, examples of enzyme surface-display strategies illustrate the feasibility of this approach for acetaldehyde detection [120,121].

Acetic acid can be formed at any time from the beginning of wine production (in grapes) until the final product (bottled wine), as a bacterial or yeast metabolite. It can be produced before alcoholic fermentation by bacterial spoilage in *Botrytis cinerea*-infected grapes, or by *Acetobacter* species [49], or as a by-product of alcoholic fermentation by *Saccharomyces cerevisiae* and by specific yeasts, such as *Dekkera* and its anamorph *Brettanomyces*. Other apiculate wine yeasts, mainly species of *Hanseniaspora* and the anamorph *Kloeckera*, as well as wine species of the genus Candida involved in the early phase of both spontaneous and inoculated fermentations, can lead to a high acetic acid content in wine [49]. In malolactic fermentation, the decarboxylation of malic acid into lactic acid by lactic acid bacteria is associated with changes in the amino acid and volatile composition of the wine, increasing the initial volatile acidity [122]. Starter cultures of *Oenococcus oeni* produce acetate under pantothenic acid deprivation due to CoA deficiency [123]. Acetic acid is another critical fermentation by-product that, when present in excess, can lead to significant losses in wine quality [49]. This metabolite was initially addressed using two biosensing strategies, both of which employed the *HAA1* promoter in *S. cerevisiae*. The first consisted of a fluorescence-based biosensor in which the promoter regulated the expression of TurboRed fluorescent protein (tRFP), enabling a concentration-dependent fluorescent response to acetic acid [124]. The second approach involved the construction of a synthetic transcription factor (*sTF*) that combines the acetic-acid-responsive regulator Haa1 with the heterologous DNA-binding domain *BM3R1* from *Bacillus megaterium*, generating a biosensor capable of quantifying acetic acid over a linear dynamic range of 10–60 mM [125].

Further developments have expanded the applicability of this biosensing platform. Mormino et al. [126] adapted the *Haa1*-based biosensor for high-throughput phenotypic screening, enabling the identification of acetic-acid-sensitive strains in a CRISPRi library of *S. cerevisiae*. More recently, Blick et al. [127] refined this system by optimizing synthetic promoters and biosensor components, improving response precision and applicability in industrial biotechnology.

A summary of the yeast-based biosensors developed for the detection of these chemical contaminants and fermentation by-products is presented in Table 3.

Other metabolites, important for specific beverages, can also be monitored: (i) Bienzymatic and multi-enzyme electrochemical biosensors monitor glucose and ethanol during wine or honey wine fermentation and wort fermentation, with good agreement to HPLC/GC reference methods [128,129,130,131]; (ii) Multi-channel biosensors measure lactate and other fermentation products in wine, kvass, and fermentation broths [95]; (iii) A simple alcohol oxidase–polyaniline dipstick visually quantifies ethanol (0.001–0.8%, *v*/*v*) in beer and traditional drinks for halal certification, matching GC results [132,133]. All these examples present biosensors; however, none of them is yeast-based.

### 3.2. YBB for Spoilage Microorganisms in Fermented Beverages

Spoilage microorganisms represent a significant challenge in the production of fermented beverages; yeasts such as *Brettanomyces bruxellensis* and *Candida vini*, lactic acid bacteria (*Lactobacillus plantarum*, *L. casei*, *L. hilgardi*, *Leuconostoc mesenteroides*, *Pediococcus damnosus*, or *Oenococcus oeni*), and acetic acid bacteria (*Acetobacter* spp., *Glucanacetobacter* spp.) are known to cause spoilage and sensory degradation, highlighting the need for rapid and selective detection tools [134].

YBB can recognize pathogen molecules directly, as demonstrated by Ostrov et al. [135], who engineered *Saccharomyces cerevisiae* to express fungal G protein–coupled receptors that bind pathogen mating peptides, resulting in a lycopene color readout for direct detection of Candida and other fungal pathogens. The “Patrol Yeast” platform employs antibody–receptor chimeras and membrane yeast two-hybrid signaling to detect aflatoxins, caffeine, and enterohemorrhagic *E. coli* O157 through antigen–antibody binding [105]. Yeast surface display systems can also directly quantify mycotoxin deoxynivalenol (DON) using Fab fragments [106]. Many yeast biosensors detect environmental contaminants indirectly via stress responses or metabolic activity, including metals, endocrine disruptors, and general toxicity [59,60]. Additionally, transcription-independent designs report analytes by measuring products of yeast metabolism [57]. Fungal spoilage and toxins can be detected using specific receptors or general toxicity/metabolic-interference strategies in food control [15,60].

#### 3.2.1. YBB for *Brettanomyces* and Other Yeast Contaminants

*Brettanomyces bruxellensis,* commonly known as Brett, is a major spoilage organism in winemaking because it metabolizes hydroxycinnamic acids into volatile phenols. These compounds, including 4-ethylphenol and 4-ethylguaiacol, are responsible for off-flavors in wine typically described as earthy, medicinal, smoky, or barnyard-like [136]. Additionally, Brett can persist on surfaces such as oak barrels even after cleaning, making early detection during storage and aging crucial [137].

Early detection is vital because conventional culture methods are slow and may miss early contamination events, underscoring the need for faster, more sensitive diagnostic techniques in production settings [138]. *Brettanomyces* yeasts produce compounds such as 4-ethylphenol and 4-ethylguaiacol, which are commonly used as markers for Brett activity [139].

However, there is a poor quantitative correlation between Brett cell counts and the concentrations of these marker phenols. Consequently, enumerating cells alone does not reliably predict sensory impact [137]. Furthermore, the presence of polyphenols and ethanol in wine matrices complicates direct detection, often requiring sample preparation or enrichment steps for many assays [140,141]. Some molecular and instrumental methods can be relatively expensive or may require specialized equipment that is not universally accessible to small producers.

Regarding whole-cell and synthetic biology sensors for Brett detection in wine and other fermented beverages, the literature provides insufficient evidence for engineered yeast whole-cell biosensors or genetic circuit-based detection of *Brettanomyces*; none of the reviewed studies report using genetically modified *Saccharomyces* or other live yeast as sensing/reporting elements. Nevertheless, several biosensor platforms and detection mechanisms have been developed for the early detection of *Brettanomyces*, encompassing a wide range of analytical principles. Quartz Crystal Microbalance (QCM) genosensors, for instance, enable the detection of DNA hybridization events by immobilizing short single-stranded DNA or extracted genomic DNA onto surface-bound probes, generating real-time signals based on mass changes. These systems have been successfully applied to the detection of *Brettanomyces* DNA in wine matrices [137].

Electrochemical disposable sensors have also been reported, notably those employing antibody- or lectin-functionalized magnetic nanoparticles to selectively capture yeast cells, which are subsequently quantified amperometrically using screen-printed electrodes. These platforms have demonstrated detection ranges of 10–10^6^ CFU/mL, with limits of detection of approximately 6–8 CFU/mL in wine or buffer solutions [141].

Interdigitated microelectrode impedance sensors represent another label-free approach, relying on impedance spectroscopy to monitor microbial growth and biofilm formation directly on electrode surfaces. This technique enables early detection of spoilage yeasts and has been shown to yield enhanced signal responses under dynamic conditions, such as continuous stirring [142].

Flow cytometry is a rapid and robust alternative, enabling cytometric profiling that distinguishes *Brettanomyces* cells from other yeast species by optical properties and staining patterns, and also enables quantitative analysis of mixed microbial populations [138].

In addition, colorimetric enzymatic assays have been developed to detect metabolites associated with *Brettanomyces* activity, particularly 4-ethylphenol. These assays rely on enzyme-mediated conversion and redox-based signal generation, permitting low-level metabolite detection using relatively simple instrumentation [143,144].

Finally, molecular hybridization-based methods, such as sandwich RNA hybridization assays with colorimetric readout, offer high species specificity and practical detection limits on the order of 10^2^ CFU/mL in wine matrices, making them suitable for applied monitoring contexts [140].

*Candida* yeasts are prevalent in various fermented beverages, functioning as fermenters, spoilage organisms, or contaminants depending on the specific species and environmental conditions. Research is commonly directed toward *Candida* spp. generically, the insights are pertinent to the concerns surrounding contamination, particularly about *Candida vini*.

*Candida* spp. are routinely identified in the spontaneous fermentations of wine, cider, beer, palm wine, cereal beverages, and traditional drinks globally [145,146,147,148]. These yeasts often proliferate during the early stages of fermentation and can endure in alcoholic, acidic environments, withstanding ethanol concentrations of approximately 11–13% [149,150,151]. In traditional African and Ethiopian beverages, certain *Candida* spp., including *C. krusei*, have been documented as spoilage agents, associated with undesirable film formation, discoloration, and off-flavors [146,148,152].

Film-forming species such as *Candida* and *Pichia* pose a risk of post-fermentation contamination in cider and wine, potentially modifying the profile of aroma-active compounds and leading to the formation of surface films. Cider, in particular, is especially susceptible due to its lower alcohol content [149]. Particular *Candida* species, including *C. krusei*, *C. pelliculosa*, and *C. lipolytica*, have been recognized as primary spoilage agents in specific beverage types [146]. Notably, the contaminant Candida and associated yeasts exhibit strong adherence to winery filtration systems and may form biofilms on stainless steel surfaces, complicating sanitation efforts [153].

A review highlights that multiple *Candida* spp. utilized or found in food fermentations are opportunistic human pathogens, emphasizing that thorough safety assessments are frequently overlooked. The routine inclusion of these yeasts as starter cultures without appropriate safety screening is not advised [154]. Some beverages effectively inhibit pathogens, including *Candida albicans*, while others may promote pathogen proliferation if pH and alcohol concentrations are low or hygiene practices are inadequate [152].

The detection of *Candida* species in fermented beverages is crucial for ensuring product safety and controlling spoilage. However, most current biosensor developments focus on clinical diagnostics or general yeast detection, rather than on assays tailored specifically for beverages [155]. Existing research on yeast-based biosensors and *Candida* detection provides essential components that could be repurposed for applications in fermented drinks.

Recent advancements include electrochemical impedimetric biosensors utilizing lectins such as Concanavalin A (ConA) and Wheat Germ Agglutinin (WGA), which can identify and differentiate multiple *Candida* species (including *C. albicans*, *C. tropicalis*, *C. krusei*, and *C. parapsilosis*) with a limit of detection (LOD) around 10^2^ CFU/mL by observing variations in charge-transfer resistance [153].

Additionally, a peptide-modified TiO_2_ nanoparticle impedimetric sensor, known as CLAVMO, has demonstrated the ability to discriminate *Candida* species and their ploidy levels, achieving a remarkable LOD of 2–3 CFU/mL within a 20 min assay time [156]. Other methodologies, such as surface plasmon resonance and nanoporous alumina platforms, have been functionalized with mannan or DNA probes, enabling sensitive and selective detection of *Candida* and facilitating immunoreactivity testing [157,158].

A recent review has highlighted various modalities, including electrochemical, optical, nanobiosensor, and CRISPR-enabled techniques, for rapid *Candida* diagnostics [159]. Despite these advancements, a fully developed yeast-based biosensor for the specific detection of *Candida* spp. in fermented beverages has not yet been reported. Nonetheless, existing electrochemical and whole-cell yeast biosensor platforms already offer highly sensitive, species-level detection of *Candida* in diverse matrices. The next logical step is to adapt lectin, peptide, or pheromone-GPCR recognition elements into robust yeast biosensor formats and validate these systems in real beverage environments. So, future work must focus on fermentation-compatible whole-cell yeast biosensors (GPCR/colorimetric) [59,135,160]—using *Candida*-specific ligands such as lectins, AMPs, pheromone-derived peptides, and DNA probes [153,156,157,158]—and on their direct validation in beverages where *Candida* spp. are prevalent [161,162].

#### 3.2.2. YBB for Lactic Acid Bacteria and Acetic Acid Bacteria

Monitoring lactic acid bacteria (LAB) and acetic acid bacteria (AAB) is essential for flavor enhancement, safety assurance, and process control during beverage fermentation. Recent advancements have integrated yeast biology with Raman/SERS techniques and whole-cell biosensors to achieve high-sensitivity detection of organic acids and microbial activity.

Engineered strains of *Saccharomyces cerevisiae* that incorporate acetic-acid-responsive transcription factors, such as Haa1 or acetic-acid-inducible promoters, enable the quantification of acetic acid through the expression of fluorescent proteins. These biosensors exhibit a linear response for acetic acid concentrations ranging from approximately 10 to 60 mM and have shown practical application in analyzing real samples and conducting high-throughput screening [124,125,126]. Although these systems do not provide specificity for AAB, they do provide a measure of acetic acid production by these bacteria.

Furthermore, whole-cell biosensors for both L- and D-lactic acid have been developed in *Escherichia coli* and *Pseudomonas putida*, facilitating the monitoring and optimization of lactic acid fermentation processes by *Lactobacillus* species [163]. An enzymatic lactate oxidase electrode sensor has also been fine-tuned to quantify lactic acid in beer within a concentration range of 10 to 2700 μM, demonstrating impressive stability [164]. This sensor targets lactic acid directly rather than LAB cell enumeration.

Additionally, the electrochemical dynamics associated with *S. cerevisiae* fermentation, particularly pressure and impedance measurements, have been utilized to create a cost-effective microbiological sensor capable of detecting environmental perturbations [165]. This suggests a potential approach for utilizing yeast response profiles to indirectly infer the presence of inhibitory metabolites produced by LAB and AAB.

Recent SERS/Raman-based methodologies have proven effective for the rapid isolation and classification of LAB and yeasts in fermented foods at concentrations as low as 10 CFU/mL, achieving over 98% recovery and 100% classification accuracy using machine learning [166,167]. While these techniques are powerful for monitoring LAB-yeast consortia, they are not exclusively yeast-based.

BN-doped carbon dots have been developed to label live and dead LAB and yeast cells with distinct fluorescence signatures, enabling rapid assessment of cell viability and activity [168]. In Table 4, we compare available biosensor and spectroscopic tools for LAB/AAB-related monitoring, considering the target signal and their relevance to beverages.

Recent yeast biosensors primarily detect acetic acid instead of directly identifying lactic acid bacteria (LAB) or acetic acid bacteria (AAB). The complexity of fermented beverage matrices poses significant challenges for specificity and dynamic range [124,125,169]. A promising avenue for improvement involves engineering yeast strains that express reporter genes under LAB/AAB-responsive promoters and integrating these with advanced techniques, such as microfluidics or Raman/SERS-based classification. None yet provides a turnkey “yeast biosensor that directly detects LAB and AAB cells” in beverages, but combining yeast acetic acid sensors, lactic acid biosensors, and rapid spectroscopic classification provides a practical toolkit and a clear engineering path toward such targeted yeast-based biosensors.

Current research has produced effective yeast whole-cell sensors for acetic acid and complementary biosensors for lactic acid, as well as SERS-based and fluorescent methodologies for the rapid detection of lactic acid bacteria (LAB) and yeast. Despite promising developments, most yeast-based biosensors remain at the laboratory or early-prototype stage, with only a limited number having been evaluated in real industrial environments [59,135,138,170].

Fermented beverages matrix complexity, need for low detection limits (≤10 CFU/mL), device robustness, and on-site usability [59,138,141,171], making the development of this kind of sensor a challenge.

## 4. Final Remarks

Yeast-based biosensors often use indirect, metabolism-related signals, but there is clear evidence for direct detection of pathogen-specific peptides, toxins, and even whole bacteria via receptor or antibody binding, so detection is not limited to excreted metabolic products.

Although yeast-based biosensors have demonstrated considerable potential for monitoring safety- and quality-related parameters in fermented beverages, their current state of development reveals an apparent discrepancy between conceptual promise and practical implementation. As highlighted throughout this review, the majority of yeast-based biosensor systems remain confined to controlled laboratory conditions, with limited validation in real beverage matrices. The chemical and microbiological complexity of fermented beverages—characterized by variable ethanol content, low pH, high sugar concentrations, and abundant interfering compounds—continues to represent a significant obstacle to sensor robustness, specificity, and reproducibility.

A further limitation lies in the uneven maturity of sensing targets. While yeast biosensors for heavy metals and selected fermentation by-products, such as acetic acid, are relatively well established, critical hazards, including biogenic amines and key spoilage microorganisms, remain largely unaddressed by yeast-based whole-cell sensing strategies. In particular, the absence of engineered yeast biosensors specifically designed for *Brettanomyces* or *Candida* spp. detection represents a notable gap, especially given the industrial relevance of these contaminants. Current detection approaches for these microorganisms rely predominantly on molecular, immunological, or electrochemical platforms that, although effective, lack the biological integration and self-reporting advantages inherent to whole-cell yeast systems.

From a technological perspective, issues related to detection limits, response time, long-term stability, and calibration under fluctuating fermentation conditions have not yet been satisfactorily resolved. Growth inhibition is especially problematic as a sensing mechanism because stressors can elicit similar cellular responses. Without appropriate controls, it may be difficult to distinguish between target-specific effects and nonspecific toxicity. Therefore, reliance on growth inhibition alone as a detection signal should be approached with caution, and complementary readouts are often necessary to improve specificity. In addition, accurate quantification of biosensor responses is inherently dependent on cell concentration. Variations in cell density, growth rate, or viability can significantly affect signal intensity and reproducibility, leading to over- or underestimation of analyte concentrations, even when the target is present at the same level. Standardization of inoculum size, normalization of signal output (e.g., to optical density or total protein), and calibration using well-defined cell densities are therefore critical for reliable quantification.

Moreover, regulatory acceptance, biosafety concerns associated with genetically modified organisms, and the lack of standardized validation protocols further constrain the deployment of yeast-based biosensors at an industrial scale. These challenges underscore the need for more rigorous benchmarking against conventional analytical methods and for systematic performance assessment under real production environments.

Indeed, under current European regulations, there are great limitations on the use of genetically modified organisms (GMOs) in food production. This fact limits the direct application of genetically engineered whole-cell biosensors (YBBs) in food matrices, which might delay their development for food production systems, raising the question of whether continued technological advancements in YBBs justify the associated research and development investments. Even so, these advancements in YBB design may still hold substantial value beyond direct food applications, for instance, to be employed in upstream process monitoring, environmental surveillance, laboratory-based food safety testing, or as disposable analytical tools where containment prevents GMO release.

Furthermore, regulatory landscapes are not static and may evolve alongside technological innovations, particularly if biosensors can demonstrate clear benefits in safety, traceability, and sustainability while ensuring effective biological containment. Consequently, while current regulations limit immediate implementation in European food production, research into YBB advancements remains scientifically justified, provided that realistic deployment pathways and alternative applications are clearly articulated.

Future progress in this field will depend on moving beyond proof-of-concept designs toward application-driven sensor engineering. This includes the development of fermentation-compatible genetic circuits, improved signal amplification strategies, and sensor architectures capable of maintaining performance in complex matrices. Equally important is the integration of yeast biosensors into modular, portable, and user-friendly devices suitable for on-site monitoring. Without addressing these critical limitations, yeast-based biosensors are likely to remain valuable research tools rather than becoming routine components of quality and safety management systems in the fermented beverage industry.

## Figures and Tables

**Table 1:** Contaminants and toxins in fermented beverages: adverse health effects and maximum limits.

Category	Contaminant/Toxin	Adverse Health Effects	Maximum Limit
Mycotoxins	Ochratoxin A	Nephrotoxic, hepatotoxic, teratogenic, immunotoxic, and carcinogenic, also associated with protein synthesis inhibition, DNA damage, oxidative stress, and multi-organ toxicity [30]	2 μg/kg [31]
	Patulin	Hepatotoxic, nephrotoxic, immunosuppressive, embryotoxic; acute exposure linked to gastrointestinal, neurological, and pulmonary effects [32]	50 μg/kg [31]
Biogenic amines	Histamine	Hypotension, flushing, headache, gastrointestinal disturbances, cardiovascular and neurological effects, and respiratory complications [33]	No maximum legal limit established; reported levels 0.1–5.0 mg/L in beer; <LOQ-28.1 mg/L in red wine; 0.3 mg/L in white wine [34,35,36]
	Tyramine	Hypertensive crises, migraines, nausea, vomiting, neurological disorders, hypertension, and respiratory effects [37]	No maximum legal limit established; reported levels 0.4–31.7 mg/L and 0.10–84.88 mg/L in beer; 1.8–4.3 mg/L in red wine; 1.0 mg/L in white wine [34,36,38,39]
	Putrescine	Potentiates the toxicity of other amines; potential carcinogenic precursor; cardiovascular alterations [40]	No maximum legal limit established; reported levels 2.18–46.14 mg/L in beer; 1.8–82.1 mg/L in red wine; 0.3 mg/L in white wine [32,38,41]
Heavy Metals	Cadmium (Cd)	Carcinogenic and toxic effects: cancer risk, renal dysfunction, neurological and cardiovascular disorders [7]	0.01 mg/L [42]
	Lead (Pb)		0.20 mg/kg (harvests 2001–2015); 0.15 mg/kg (2016–2021); 0.10 mg/kg (from 2022 onwards) [31]
	Arsenic (As)		0.2 mg/L [42]
	Boron (as boric acid)		80 mg/L [42]
	Bromide (Br^−^)		1 mg/L (up to 3 mg/L in wines from brackish subsoil) [42]
	Copper (Cu)		1 mg/L (2 mg/L for liqueur wines) [42]
	Fluoride (F^−^)		1 mg/L (3 mg/L if cryolite-treated vineyards) [42]
	Silver (from silver chloride)		<0.1 mg/L [42]
	Sodium (excess)		80 mg/L [42]
	Zinc (Zn)		5 mg/L [42]
Organic contaminants	Diethylene glycol		10 mg/L (limit of quantification) [42]
	Ethylene glycol (Ethanediol)		10 mg/L [42]
	Methanol		400 mg/L (red wines); 250 mg/L (white and rosé wines) [42]
Pathogenic Microorganisms	*Escherichia coli*	Indicative of fecal contamination; some strains are pathogenic, causing severe stomach cramps, bloody diarrhea, and vomiting [6]	Absence in 25 mL [43]
	*Listeria monocytogenes*	Severe illness (meningitis, sepsis, miscarriage, neonatal infection); mild gastrointestinal symptoms in non-invasive cases [44]	Absence in 25 mL [43]
Preservatives/Additives	Sulphites (SO_2_)	Allergic reactions, asthma, gastrointestinal symptoms [45]	150–400 mg/L depending on wine type and residual sugar [42,46]
	Sorbic acid	Generally safe but may cause allergic reactions or off-flavors if above limits [47]	200 mg/L [42]
	Natamycin	Generally safe; poorly absorbed; high doses may cause gastrointestinal irritation, nausea, and diarrhea [48]	5 μg/L (provisional decision limit) [42]
Fermentation by-products	Acetic acid (volatile acidity)	Excess levels cause an undesirable vinegar-like flavor, which is also indicative of spoilage fermentation [49]	1.2 g/L (20 mEq/L) [42,46]
	Acetaldehyde	Possibly carcinogenic (IARC Group 2B); facial flushing, nausea, vomiting, tachycardia, hypotension; hepatotoxic and neurotoxic effects [50]	No maximum legal limit established. Typical concentration ranges in fermented beverages: Beer: 0–63 mg/L Wine: 0–211 mg/L Fortified wines: 12–800 mg/L Spirits: 0–1159 mg/L [51] Sensory threshold in wines: approx. 100–125 mg/L (free acetaldehyde) [52]
Endocrine-disrupting compounds (EDCs)	BPA, phthalates, PFAS (such as Teflon), pesticides	Obesity, diabetes, and fertility issues	No maximum legal limit established or quantified. Found in food, packaging, and water [21,53]
	Flame retardants, alkylphenols	Neurodevelopment, hormone-sensitive cancers	No maximum legal limit established or quantified. Consumer products, dust [21,53]
	Natural estrogens, plasticizers	Thyroid, reproductive, and metabolic effects	No maximum legal limit established or quantified. Aquatic environment [21,54]
Antibiotics	Tetracyclines, penicillins, macrolides, and others	Continual exposure to and resistance to antibiotics create an AMR problem in the food chain.	No numeric “safe antibiotic dose” in the food product itself [26,27]

**Table 2:** Main types of yeast-based biosensors, their detection principles, analytes, advantages, and limitations.

Type of Yeast-Based Biosensor	Reporter Signal/Detection Principle	Typical Analytes	Main Advantages	Limitations/Notes	Ref.
Fluorescent/ Luminescent	Expression of fluorescent or luminescent proteins such as GFP, RFP, YFP, Lux, or Luc. Detection based on fluorescence or light emission intensity.	Heavy metals, toxic compounds	High sensitivity; direct visual or instrumental readout; no substrate addition required; rapid response.	Requires excitation light (for fluorescence); luminescence depends on enzymatic activity.	[57,60,74,75,76]
Enzymatic/ Colorimetric	Enzyme-mediated reactions produce visible color or absorbance changes, often using β-galactosidase or peroxidase as reporter enzymes.	General toxic compounds, metabolic stress indicators	Simple, low-cost, suitable for visual or spectrophotometric detection.	Limited quantitative precision; possible interference from matrix effects.	[60,69,77,78]
Electrochemical/ Amperometric	Measurement of electric current, potential, or conductance variation resulting from yeast metabolic reactions or redox activity.	Heavy metals, volatile organic acids	High sensitivity; portable and compatible with low-cost devices; suitable for real-time or in situ monitoring.	Requires electrode calibration and stable measurement conditions.	[77,79,80]

**Table 3:** YBB developed for the detection of contaminants and fermentation by-products in fermented beverages.

Target Category	Analyte	Biosensor System/Reporter Gene	Yeast Species	Main Application/Advantage	Ref.
Heavy metals	Cu^2+^	*Cup1* promoter—GFP	*S. cerevisiae*	Highly selective copper detection	[91]
		*Cup1* promoter—luciferase	*S. cerevisiae*	Sensitive luminescent detection	[92]
		*CUP1* promoter—LacZ/ADE2	*S. cerevisiae*	Colorimetric/auxotrophic detection	[93]
		Engineered *Cup1* promoter + dual fluorescent reporters (*Cup2* overexpression)	*S. cerevisiae*	Broad linear range; high specificity	[94]
	Cd^2+^	*HSEO1* promoter—GFP	*S. cerevisiae*	Dose-dependent Cd detection	[95]
		*HpSEO1* promoter—GFP	*Hansenula polymorpha*	Yeast-based Cd biosensing	[95]
	Cu^2+^, Cd^2+^	Multi-organism biosensor (includes *S. cerevisiae*)	*Mixed microbes*	Dual-metal detection	[96]
Mycotoxins	T2 toxin, Verrucarin A	β-galactosidase inhibition assay	*Kluyveromyces fragilis*, *K. marxianus*	Early yeast toxin biosensors	[100,101]
	Zearalenone	Human estrogen receptor (hER)—growth-based biosensor	*S. cerevisiae*	Detects estrogenic mycotoxin activity	[102]
		hER + firefly luciferase reporter	*S. cerevisiae*	Sensitive bioluminescent detection	[103]
	Aflatoxin B_1_	Reporter gene induction (GFP/LacZ)	*S. cerevisiae*	Yeast genotoxicity-based detection	[104]
	Aflatoxin B_1_, M_1_	β-galactosidase assay	*S. cerevisiae*	Colorimetric aflatoxin biosensor	[105]
	Deoxynivalenol (DON)	Yeast surface display (Fab antibodies)	*S. cerevisiae*	Particular DON binding	[106]
		YSD chemiluminescent biosensor	*S. cerevisiae*	DON detection in beverages	[106]
Biogenic amines	–	–	*–*	No yeast-based biosensors have been reported	-
Fermentation by-products	Acetaldehyde	Enzyme surface display (acetaldehyde dehydrogenase)	*E. coli + S. cerevisiae*	Detection in wine	[120]
		Optical biosensor based on enzyme surface display	*E. coli + S. cerevisiae*	Acetaldehyde detection in beverages	[121]
	Acetic acid	*Haa1* promoter—tRFP	*S. cerevisiae*	Monitoring volatile acidity	[124]
		*Haa1*–*BM3R1* synthetic transcription factor	*S. cerevisiae*	Quantitative metabolic sensing	[125]
		*Haa1*-based CRISPRi screening biosensor	*S. cerevisiae*	Identification of acetic-acid-sensitive strains	[126]
		Optimized *Haa1* synthetic promoter biosensor	*S. cerevisiae*	Improved precision for industrial biotechnology	[127]

**Table 4:** Comparison of biosensor and spectroscopic tools for LAB/AAB-related monitoring.

Approach/Host	Target Signal	Relevance to LAB/AAB Beverages	Ref.
Yeast Haa1 TF biosensor	Acetic acid (fluorescence)	Indirect AAB monitoring, strain screening	[124,125,126]
L/D-lactate whole-cell (bacteria)	Lactic acid	LAB fermentation monitoring, optimization	[163]
Lactate oxidase electrode	Lactic acid in beer	Inline/at-line beer quality control	[164]
SERS + magnetic enrichment	LAB & yeast cells	Rapid cell-level monitoring in fermentations	[166,167]
BN-CD fluorescence	LAB/yeast viability	Fast activity readout in starters	[168]

## Data Availability

No new data were created or analyzed in this study.

## Abbreviations

The following abbreviations are used in this manuscript:

AAB	Acetic Acid Bacteria
ADE2	A gene involved in the adenine biosynthesis pathway in *Saccharomyces cerevisiae*; when used as a reporter, ADE2 expression enables visual detection via colony color changes (white vs. red)
AFB1	Aflatoxin B1, a highly toxic and carcinogenic mycotoxin produced mainly by *Aspergillus* species
AFM1	Aflatoxin M1, a hydroxylated metabolite of aflatoxin B1, can appear in milk and fermented products
AMR	Antimicrobial resistance
ARGs	Antibiotic-resistant bacteria and genes
BM3R1	DNA-binding domain derived from the BM3R1 regulator of *Bacillus megaterium*; used heterologously to confer specific DNA-binding capability in synthetic transcription factors
BN	Boron nitride—a nanomaterial (often hexagonal boron nitride, h-BN) used in sensing applications due to its chemical stability, optical properties, and ability to enhance fluorescence signals
BPA	Bisphenol A
CD	Carbon dots—nanoscale carbon-based fluorescent nanoparticles known for strong photoluminescence, high stability, and low toxicity
CFU	Colony-forming units
ConA	Concanavalin A
CRISPRi	CRISPR interference—a gene-silencing technology that uses a catalytically inactive Cas protein to repress transcription without cutting DNA
CUP1	Copper Metallothionein 1 promoter—a copper-inducible promoter in *S. cerevisiae*
Cup2	Copper-responsive transcriptional activator (also known as Ace1) that regulates CUP1 expression
DNA	Deoxyribonucleic acid
DON	Deoxynivalenol—a trichothecene mycotoxin also known as vomitoxin
EDCs	Endocrine-disrupting compounds
ELISA	Enzyme-linked immunosorbent assay
Fab	Fragment antigen-binding—the antigen-binding region of an antibody, consisting of one constant and one variable domain from both the heavy and light chains
Fab@YSD	Fab fragments displayed on the surface of yeast cells using YSD technology
FACS	Fluorescence-Activated Cell Sorting—a flow-cytometry-based method that sorts cells according to fluorescent signals
FDA	Food and Drug Administration
GC-MS	Gas chromatography coupled with mass spectrometry
GFP	Green Fluorescent Protein
GPCR	G protein-coupled receptor—a large family of membrane receptors that detect extracellular signals (such as pheromones) and activate intracellular signaling cascades
GRAS	Generally recognized as safe
Haa1	Acetic-acid-responsive transcriptional regulator in *S. cerevisiae* that mediates cellular tolerance to acetic acid
HAA1	High Acetic Acid Tolerance 1—a transcription factor in *Saccharomyces cerevisiae* that regulates genes involved in the cellular response to acetic acid; the HAA1 promoter is activated under acetic acid stress
hER	human estrogen receptor
HPLC	High-performance liquid chromatography
HpSEO1 promoter	The SEO1 gene promoter from *Hansenula polymorpha* (the prefix Hp denotes the species), often referring to a defined promoter fragment (e.g., 500 bp) used for cadmium-inducible expression
HSEO1/HpSEO1	SEO1 gene promoter from *Hansenula polymorpha*; HpSEO1 refers explicitly to the 500 bp promoter fragment used in reporter constructs
HSEO1 promoter	The promoter region of the SEO1 gene from yeast is responsive to cadmium and other heavy metals. It has been used to drive reporter gene expression in metal-responsive biosensors
IARC	International Agency for Research on Cancer
IUPAC	International Union of Pure and Applied Chemistry
LAB	Lactic Acid Bacteria
LACZ	Gene encoding β-galactosidase, widely used as a colorimetric reporter gene
LacZ	A reporter gene encoding β-galactosidase, an enzyme commonly used in colorimetric assays (e.g., X-gal hydrolysis) to report promoter activity
LOQ	Limit of quantification
Luc	Luciferase (most commonly firefly luciferase), which emits light in the presence of a substrate such as luciferin
Lux	Luciferase system encoded by the lux operon (typically bacterial luciferase that produces light autonomously
LuxCDABE	Bacterial luciferase (lux) gene cassette consists of five genes (CDABE) whose protein products synergistically generate bioluminescent light signals exclusive of supplementary substrate additions or exogenous manipulations
MACS	Magnetic-Activated Cell Sorting—a technique that uses magnetic beads to isolate cells expressing specific surface markers
mCherry	A basic (constitutively fluorescent) red fluorescent protein was published in 2004, derived from *Discosoma* sp., reported to be a very rapidly maturing monomer with low acid sensitivity
NADH	Nicotinamide adenine dinucleotide plus hydrogen
QCM	Quartz Crystal Microbalance
Raman	Raman spectroscopy—an analytical technique based on inelastic scattering of light, providing molecular “fingerprint” information about chemical compounds
RFP	Red Fluorescent Protein
SERS	Surface-Enhanced Raman Scattering—an advanced form of Raman spectroscopy in which the Raman signal is greatly amplified by molecules adsorbed onto nanostructured metallic surfaces (typically silver or gold)
SPiDER-βGal	A reagent that detects β-galactosidase and possesses high cell-permeability and intracellular retentivity
sTF	Synthetic Transcription Factor—an engineered regulatory protein that combines functional domains (e.g., DNA-binding and activation domains) to control gene expression in response to a specific stimulus
TF	Transcription factor
PFAS	Per- and polyfluoroalkyl substances (informally called “forever chemicals”) are a group of synthetic organofluorine chemical compounds that have multiple fluorine atoms attached to an alkyl chain
WGA	Wheat Germ Agglutinin
YAS	Yeast Androgen Screen
YES	Yeast Estrogen Screen
YBB	Yeast-based biosensors
YFP	Yellow Fluorescent Protein
YSD	Yeast Surface Display—a technology in which proteins (e.g., antibodies) are displayed on the surface of yeast cells
ZON	Zearalenone
